# The Role of Glutamine Synthetase in Regulating Ammonium Assimilation and Iron-Only Nitrogenase Expression in a Photosynthetic Diazotroph

**DOI:** 10.1128/spectrum.04953-22

**Published:** 2023-03-27

**Authors:** Mingyue Jiang, Dahe Zhao, Lu Huang, Yan Zeng, Jingfang Liu, Hua Xiang, Yanning Zheng

**Affiliations:** a State Key Laboratory of Microbial Resources, Institute of Microbiology, Chinese Academy of Sciences, Beijing, China; b College of Life Science, University of Chinese Academy of Sciences, Beijing, China; c Institutional Center for Shared Technologies and Facilities, Institute of Microbiology, Chinese Academy of Sciences, Beijing, China; University of Minnesota Twin Cities

**Keywords:** glutamine synthetase, nitrogenase, gene expression/regulation, *Rhodopseudomonas palustris*, climate change microorganisms

## Abstract

Glutamine synthetase (GS) is responsible for the ammonium assimilation into glutamine, which serves as an important nitrogen donor for the synthesis of biomolecules and also plays a key role in regulating the nitrogen fixation catalyzed by nitrogenase. Rhodopseudomonas palustris, whose genome encodes 4 putative GSs and 3 nitrogenases, is an attractive photosynthetic diazotroph for studies of nitrogenase regulation, as it can produce the powerful greenhouse gas (methane) by iron-only (Fe-only) nitrogenase using light energy. However, the primary GS enzyme for ammonium assimilation and its role in nitrogenase regulation remain elusive in R. palustris. Here, we show that GlnA1, whose activity is finely regulated by reversible adenylylation/deadenylylation of Tyr^398^ residue, is primarily responsible for ammonium assimilation as the preferred GS in R. palustris. The inactivation of GlnA1 makes R. palustris shift to use the alternative GlnA2 for ammonium assimilation, resulting in the expression of Fe-only nitrogenase even in the presence of ammonium. We present a model, showing how R. palustris responds to ammonium availability and further regulates the expression of Fe-only nitrogenase. These data may contribute to the design of promising strategies for a better control of greenhouse gas emissions.

**IMPORTANCE** The photosynthetic diazotrophs, such as Rhodopseudomonas palustris, can utilize light energy to drive the conversion of carbon dioxide (CO_2_) to a much more powerful greenhouse gas methane (CH_4_) by Fe-only nitrogenase, which is strictly regulated in response to the ammonium, a substrate of glutamine synthetase for the biosynthesis of glutamine. However, the primary glutamine synthetase for ammonium assimilation and its role in nitrogenase regulation remain unclear in R. palustris. This study shows that GlnA1 is the primary glutamine synthetase for ammonium assimilation, and also plays a key role in Fe-only nitrogenase regulation in R. palustris. For the first time, a R. palustris mutant capable of expressing Fe-only nitrogenase even in the presence of ammonium is obtained by inactivation of GlnA1. A better understanding of the Fe-only nitrogenase regulation achieved in this study provide us with new insights into the efficient control of CH_4_ emissions.

## INTRODUCTION

**T**he assimilation of ammonium into glutamine and glutamate, which further serve as the nitrogen donors for the biosynthesis of key biomolecules, is of great importance in the nitrogen metabolism of all organisms (1–3). The 2 reactions for ammonium assimilation are catalyzed by glutamine synthetase (GS) and glutamate dehydrogenase (GDH), which are responsible for the incorporation of ammonia into glutamine and glutamate, respectively. GS enzyme, which can be divided into 4 categories (GSI, GSII, GSIII, and GlnT) and forms an ammonium assimilation cycle together with glutamine 2-oxoglutarate aminotransferase (GOGAT) (4, 5), has a higher substrate affinity for ammonium in Escherichia coli when compared with GDH (3, 6, 7). GS catalyzes an energy-intensive reaction that converts glutamate and ammonia to glutamine by consuming about 15% of the ATP requirement of the cells (3). Therefore, the activity of GS is strictly regulated to avoid the unnecessary consumption of energy by sensing ammonium availability. The regulation of GS activity mainly occurs at the post-translational level. GlnE, a bifunctional enzyme consisting of an adenylylating domain and a deadenylylating domain, is responsible for the adenylylation and deadenylylation of GS in response to ammonium availability. It functions as an adenylylating enzyme to inactivate GS when the nitrogen source is abundant. In contrast, when the nitrogen source is limited, GlnE acts as a deadenylylating enzyme to restore the activity of GS.

As a product of GS, glutamine plays a key role in regulating the expression of nitrogenase, which catalyzes the conversion of nitrogen gas to ammonia in a process called biological nitrogen fixation that contributes 50% of the fixed nitrogen to Earth. There are 3 homologous nitrogenases that are known as the molybdenum nitrogenase (Mo nitrogenase), the vanadium nitrogenase (V nitrogenase), and the iron-only nitrogenase (Fe-only nitrogenase), each differentiated by the metal composition in the active site cofactor (8). Just as how ammonium regulates the activity of GS, nitrogenase expression also responds to the availability of ammonium. In addition, diazotrophs preferentially express Mo nitrogenase when Mo is available, with V nitrogenase or Fe-only nitrogenase only expressed in situations where Mo is depleted. The photosynthetic diazotroph Rhodopseudomonas palustris, which can express all 3 types of nitrogenases, has received increasing attentions as it can make use of light energy to produce the powerful greenhouse gas methane (CH_4_) by Fe-only nitrogenase (9, 10). However, the physiological roles of the GS enzymes remain unknown in the nitrogen-fixing R. palustris (11).

Here, we sought to figure out the preferred GS for ammonium assimilation and its role in Fe-only nitrogenase regulation in R. palustris. We show that in R. palustris GlnA1 (GSI) is the primary GS for the assimilation of ammonium into glutamine, and GlnA2 (GSII) is the back-up enzyme that is deployed in situations where GlnA1 is absent. We also present evidence that GlnA1 plays a key role in Fe-only nitrogenase regulation in response to ammonium availability.

## RESULTS

### Four GS isoenzymes present in R. palustris fall into 3 categories.

**FIG 1:**
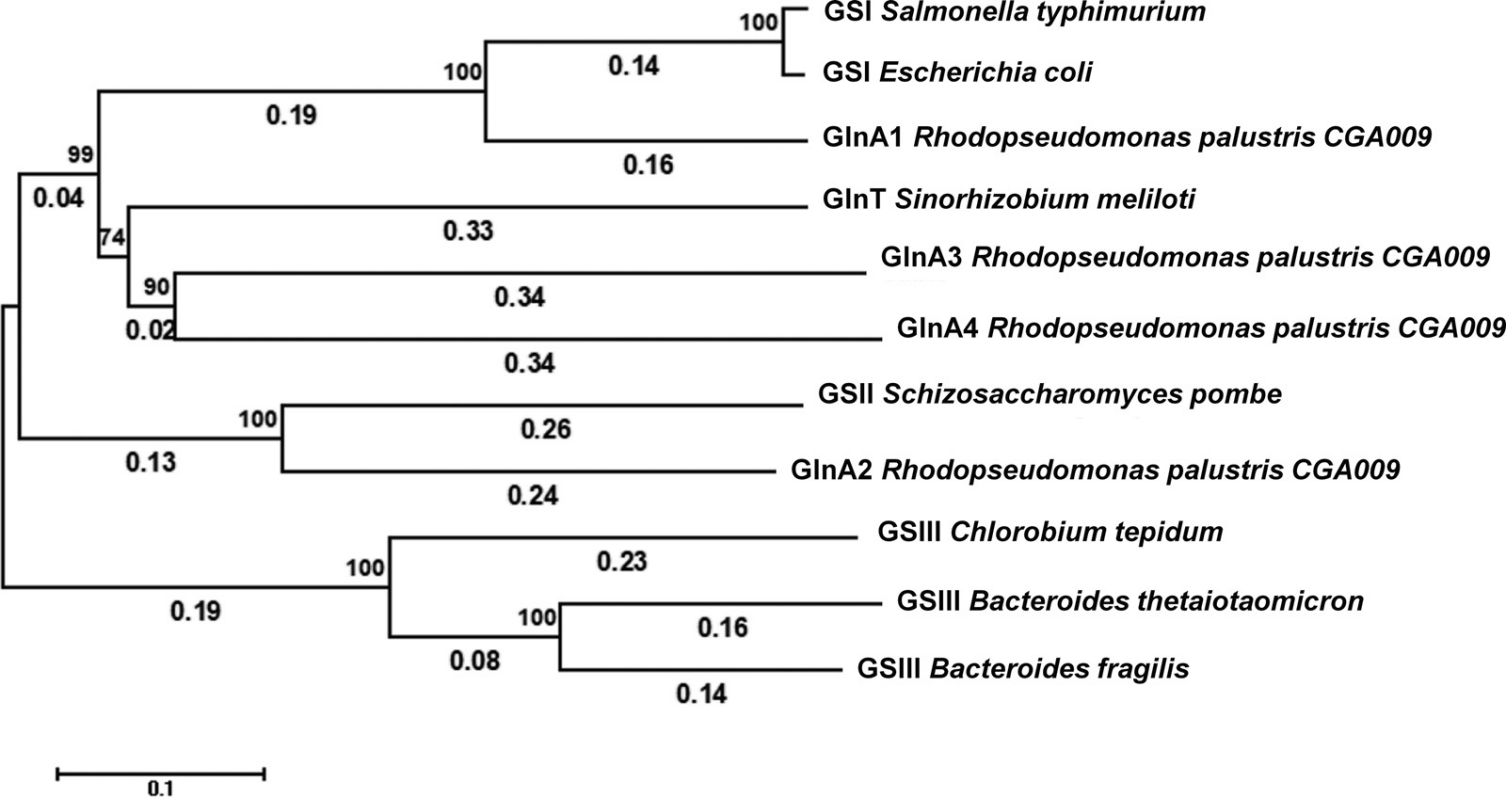
Phylogenetic analyses indicate that the 4 GS isoenzymes present in R. palustris CGA009 fall into 3 categories. GS enzymes, which catalyze the conversion of glutamate and ammonia to glutamine, were classified based on the phylogenetic relationship with canonical reference sequences. The reference sequences of the 4 GS classes are as follows: (GSI) Salmonella *typhimurium* LT2 (NP_462887.1) and E. coli MG1655 (NP_418306.1); (GSII) Schizosaccharomyces pombe ASM294v2 (NP_593400.1); (GSIII) Chlorobium tepidum TLS (WP_010933078.1), Bacteroides thetaiotaomicron ASM1413175v1 (WP_008765057.1), and B. fragilis ASM1688992v1 (WP_008768008.1); (GST) Sinorhizobium meliloti 2011 (WP_003532529.1) (Table S2).

The R. palustris genome encodes 4 putative GS enzymes: GlnA1 (Rpa2967), GlnA2 (Rpa4209), GlnA3 (Rpa1401), and GlnA4 (Rpa0984) (Table S1). Phylogenetic analyses indicated that the 4 GS enzymes fell into 3 categories (Fig. 1), with GlnA1 and GlnA2 falling into class I and class II categories of GS, respectively, and both GlnA3 and GlnA4 falling into the GlnT category. The active sites of GSI that are composed of 12 identical monomers are located at the subunit interfaces (12). The unusual subunit interactions are of great importance in regulating the GS activity in response to covalent modification. Just like other GSII enzymes, GlnA2 has about 100 fewer residues than the other 3 GS enzymes (Table S1). In addition, the expression of *glnA2*, *glnA3* and *glnA4* genes significantly increased under nitrogen-fixing conditions (9), suggesting that GSII and GlnT may play a more important role in nitrogen-limited conditions.

### GlnA1 is the primary GS for ammonium assimilation in R. palustris.

**FIG 2:**
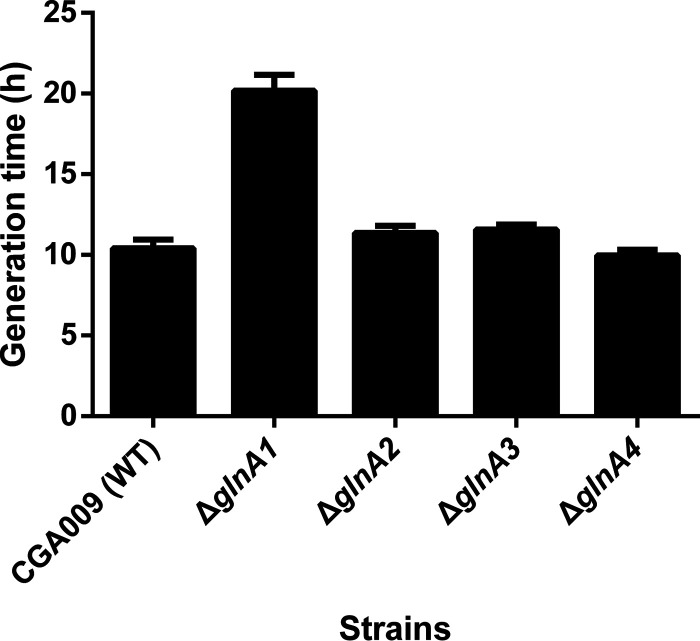
The *glnA1* deletion mutant has an obvious growth defect when grown photoheterotrophically in a defined mineral medium supplemented with ammonium (PM). The *glnA1* single deletion mutant (Δ*glnA1*) exhibited about 2 times longer generation time than the wild-type CGA009 and the other 3 *glnA* single deletion mutants (Δ*glnA2*, Δ*glnA3*, and Δ*glnA4*) (Table S3). The generation time of all strains were calculated by monitoring their OD_660_ over time. Data are the average of 3 biological replicates, and the error bars represent the s.d.

**FIG 3:**
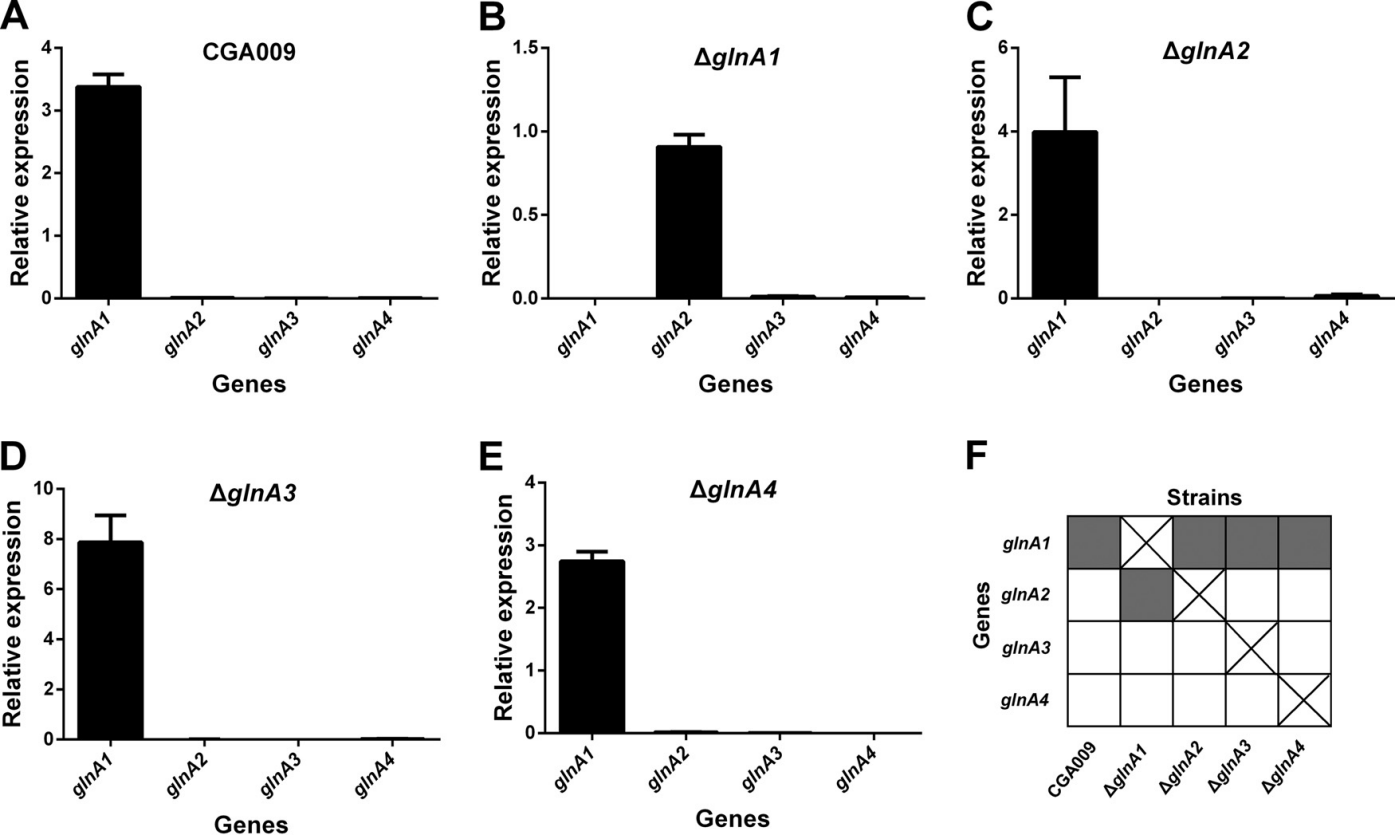
R. palustris primarily expresses *glnA1* when grown photoheterotrophically with ammonium. The qRT-PCR was employed to determine the relative transcriptional levels of *glnA1*, *glnA2*, *glnA3*, and *glnA4* genes (*glnA1-A4*) in wild-type CGA009, *glnA1*, *glnA2*, *glnA3*, or *glnA4* single deletion mutant. Except for Δ*glnA1* mutant that switches to express *glnA2* gene, the other R. palustris strains tested all express *glnA1* in PM media. The transcriptional levels of *glnA1-A4* were normalized to the transcriptional levels of *rpoD*. Data are the average of 3 biological replicates, and the error bars represent the s.d.

To examine the physiological role of different GS enzymes, we constructed in-frame deletion mutations in each of the 4 GS genes in R. palustris cells. The *glnA1* deletion mutant showed a pronounced growth defect in defined mineral medium supplemented with ammonium (PM), with a generation time about twice as long as that of the wild-type strain and 3 other *glnA* single deletion mutants (Fig. 2). To verify that GlnA1 functions as a primary GS for ammonium assimilation in R. palustris, we measured the gene expression levels of the wild-type and 4 *glnA* single deletion mutants. Except for the Δ*glnA1* mutant, all other strains primarily express *glnA1* gene. The expression of the *glnA2* gene occurred only after the deletion of the *glnA1* gene (Fig. 3). In addition, we failed to make the *glnA1* and *glnA2* double deletion mutant, suggesting that only GlnA2 can function as an alternative GS for ammonium assimilation. Altogether, these data demonstrate that GlnA1 predominates at the transcriptional level in R. palustris.

**FIG 4:**
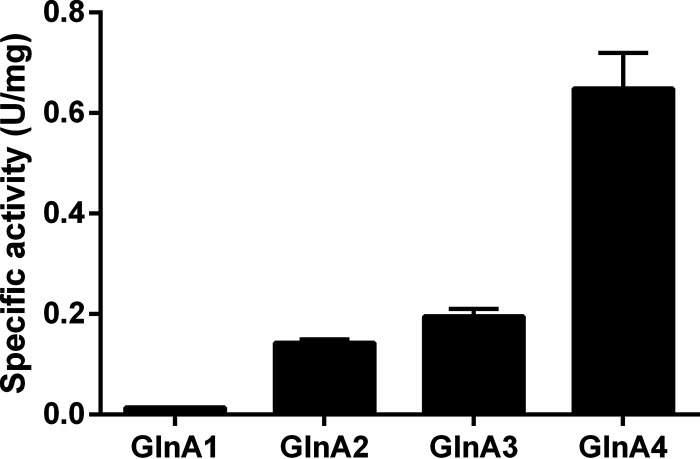
The GlnA1 purified from E. coli cells grown in nitrogen-replete conditions exhibits an extremely low activity. The His-tagged GlnA proteins were overexpressed and purified from E. coli BL21(DE3) grown in LB medium, and then the specific activities of GlnA1-A4 were determined by measuring the γ-glutamylhydroxamate synthesized from glutamine and hydroxylamine. The specific activity of the His-tagged GlnA1 was quite low when compared with that of the GlnA4. Data are the average of 3 biological replicates, and the error bars represent the s.d.

**FIG 5:**
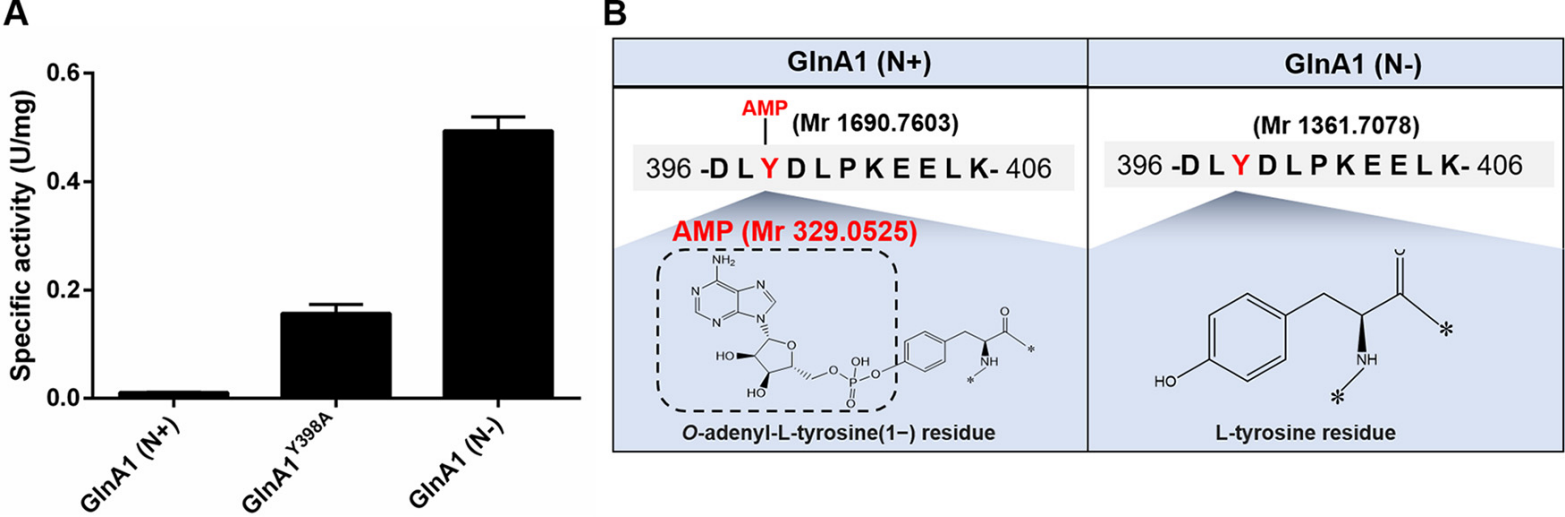
The adenylylation of Tyr^398^ residue inhibits the activity of GlnA1. (A) When E. coli cells expressing GlnA1 or its variant GlnA1^Y398A^ were grown under nitrogen-replete conditions (N+), GlnA1^Y398A^ exhibited a much higher specific activity than GlnA1. Moreover, the GlnA1 purified from cells grown under nitrogen-limited conditions (N-) also restored activity. Data are the average of 3 biological replicates and the error bars represent the s.d. (B) The nano-LC-MS/MS analysis shows that Tyr^398^ residue of GlnA1 was in adenylylated and unadenylylated states when GlnA1 was purified from E. coli cells that were grown under nitrogen-replete and nitrogen-limited conditions, respectively.

To confirm that GlnA1 can catalyze the formation of glutamine from glutamate and ammonium, the His-tagged GlnA proteins were overexpressed and purified from E. coli. The reaction conditions were optimized using the recombinant GS proteins fused with thioredoxin (Fig. S1). We were surprised to find that the specific activity of GlnA1 was extremely low whether GlnA1 was fused with thioredoxin or not, and GlnA4 exhibited a much higher specific activity than the other 3 GS enzymes (Fig. 4 and Fig. S1). Given that GlnA1 belongs to GSIs, whose activity is commonly regulated by the modification of adenylylation and deadenylylation, the severely impaired activity of GlnA1 could come from its adenylylation by the endogenous GlnE of E. coli. Therefore, we performed mass spectrometric analysis of the purified GlnA1 to determine its adenylylation state. Mass spectrometry revealed that GlnA1 was covalently modified by adenylylation of Tyr^398^ residue (Fig. S2). To promote the deadenylylation of GlnA1, E. coli cells expressing GlnA1 were transferred from LB medium to nitrogen-depleted medium before harvesting the cells. A large proportion of GlnA1 was deadenylated via mass spectrometric analysis, resulting in a 35-fold higher specific activity of GlnA1. To further verify that the deadenylylation contributed to the restored activity of GlnA1, we made a GlnA1 variant containing an amino acid substitution Y398A. The GlnA1^Y398A^ purified from E. coli cells that were grown in LB medium showed a significantly increased specific activity when compared with the wild-type GlnA1 expressed and purified under the same conditions (Fig. 5A). The nano-LC-MS/MS analysis confirmed that GlnA1 activity is regulated by a reversible adenylylation/deadenylylation modification (Fig. 5B).

### GlnA1 plays a key role in nitrogenase regulation in response to nitrogen availability.

**FIG 6:**
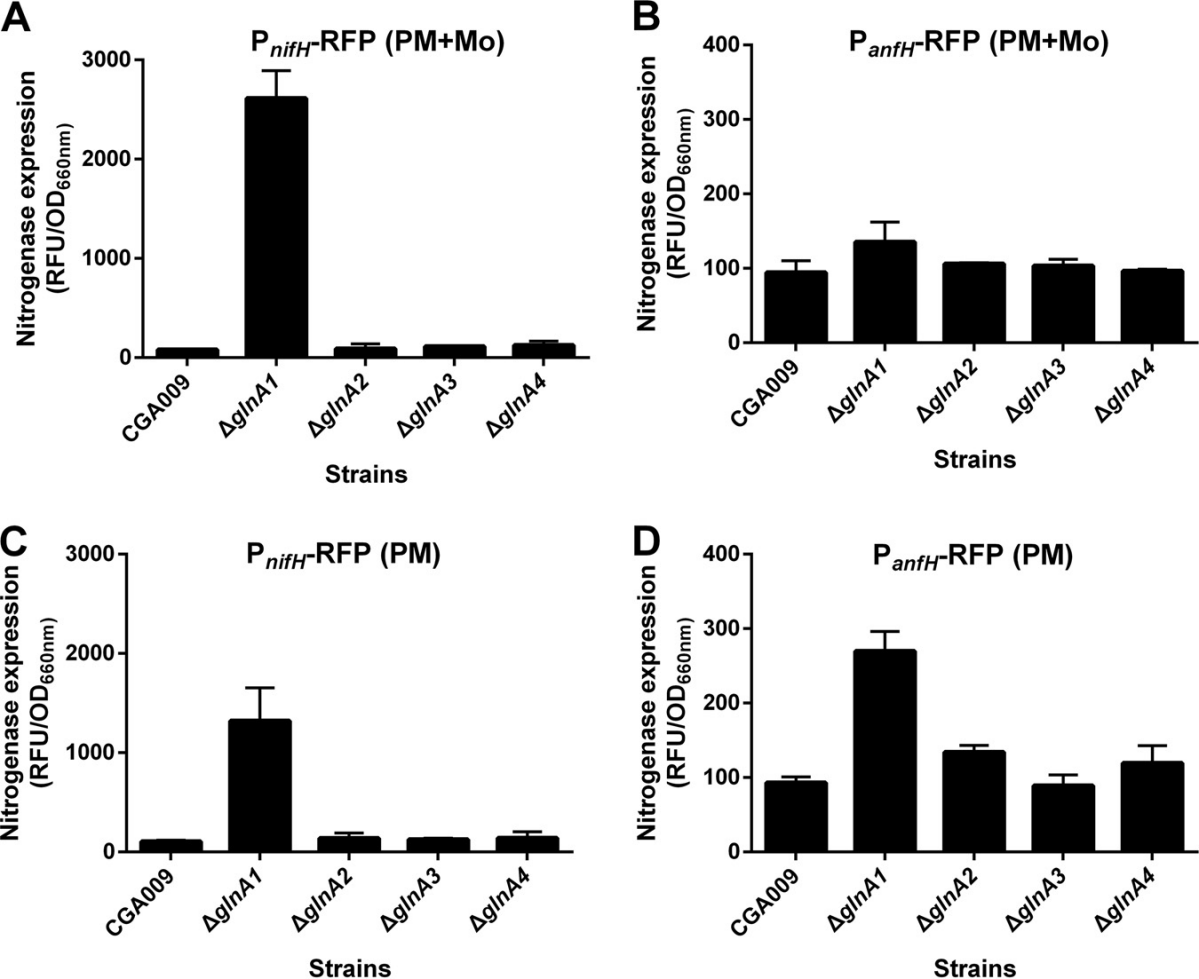
The deletion of *glnA1* gene derepresses the nitrogenase expression in R. palustris grown photoheterotrophically with ammonium. The expression of nitrogenase is subject to ammonium inhibition in wild-type R. palustris CGA009. (A and B) The deletion of *glnA1* derepressed the expression of Mo nitrogenase but not of Fe-only nitrogenase when ammonium and molybdenum are available in the medium. Mo nitrogenase is a preferred nitrogenase over the alternative Fe-only nitrogenase. The Fe-only nitrogenase is only expressed when Mo nitrogenase is unavailable or inactive. (C and D) When molybdenum was omitted from the medium, R. palustris Δ*glnA1* mutant expressed Mo nitrogenase (inactive) and Fe-only nitrogenase even in the presence of ammonium, a condition that represses expression of the Fe-only nitrogenase in wild-type R. palustris. The RFP reporter systems P*_nifH_*-RFP and P*_anfH_*-RFP were used to measure the relative expression of Mo nitrogenase and Fe-only nitrogenase, respectively. Data are the average of 3 biological replicates, and the error bars represent the s.d.

Given that glutamine is a key signal of the intracellular nitrogen status that regulates the expression of nitrogenase, GlnA1 could play an important role in nitrogenase regulation by modulating the glutamine biosynthesis in R. palustris. To examine if the deletion of *glnA* genes will induce the expression of nitrogenase in the presence of ammonium, a condition that inhibits the nitrogenase expression of the wild-type R. palustris, an RFP reporter system was firstly used to determine the levels of expression of the nitrogenase genes (13). When R. palustris Δ*glnA1* mutant was grown in PM supplemented with molybdenum, the deletion of *glnA1* induced the expression of Mo nitrogenase (NifHDK) (Fig. 6A) but not of Fe-only nitrogenase (AnfHDGK) (Fig. 6B). When molybdenum was removed from the PM medium, R. palustris Δ*glnA1* mutant expressed both Mo nitrogenase (Fig. 6C) and Fe-only nitrogenase (Fig. 6D). The deletion of *glnA2*, *glnA3*, or *glnA4* was unable to derepress the expression of Mo or Fe-only nitrogenase caused by ammonium.

**FIG 7:**
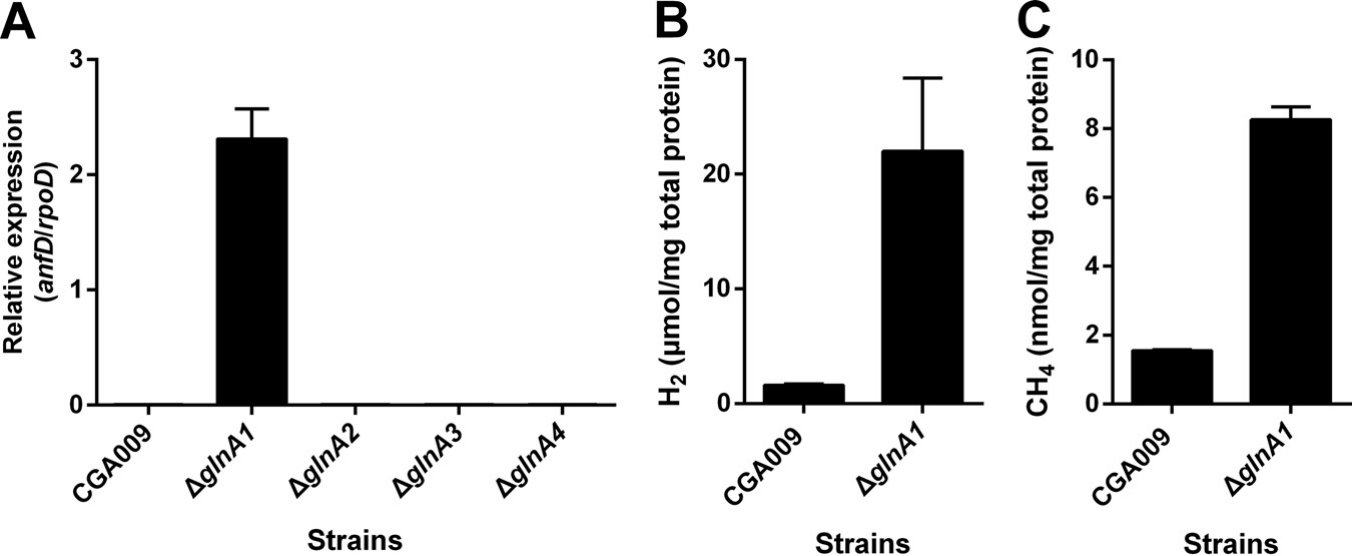
The deletion of *glnA1* makes R. palustris express an active Fe-only nitrogenase in the presence of ammonium. All R. palustris strains were grown photoheterotrophically in PM media without molybdenum. (A) The qRT-PCR analysis indicated that R. palustris Δ*glnA1* mutant grown with ammonium derepressed the expression of *anfHDGK* gene cluster that encodes Fe-only nitrogenase. (B and C) R. palustris Δ*glnA1* mutant produced H_2_ and CH_4_ in the presence of ammonium. Given that CH_4_ production is a unique property of Fe-only nitrogenase, these data suggest that an active Fe-only nitrogenase was expressed. Data are the average of 3 biological replicates, and the error bars represent the s.d.

To further verify that the expression of Fe-only nitrogenase was no longer regulated by ammonium when the *glnA1* gene was omitted from the genome of R. palustris, quantitative real-time PCR (qRT-PCR) was also employed to determine the expression levels of *anfD* gene. The qRT-PCR assay showed that the *anfD* gene was only expressed in *glnA1* deletion mutant when R. palustris cells were grown with ammonium (Fig. 7A). In addition, we also measured the H_2_ and CH_4_ production to test the activities of nitrogenases. Compared with the wild-type strain, R. palustris Δ*glnA1* mutant produced significant amounts of H_2_ and CH_4_ when grown in medium supplemented with ammonium (Fig. 7B and C), which inhibits the expression of all 3 nitrogenases in the wild-type R. palustris. These results confirm that the deletion of *glnA1* gene can induce the expression of an active Fe-only nitrogenase.

## DISCUSSION

The reduction of N_2_ to ammonia catalyzed by nitrogenase and the following ammonia assimilation catalyzed by GS or GDH are 2 important processes for the survival of the widely distributed diazotrophs when they are grown under nitrogen-limited conditions. R. palustris is a metabolically versatile photosynthetic bacterium that encodes all 3 types of nitrogenases (9, 11). Because of the absence of *gdh* gene, the conversion of glutamate and ammonia to glutamine catalyzed by GS is the only pathway for ammonia assimilation in R. palustris. Genomic analyses indicate that R. palustris encodes four GSs, but their physiological functions remain elusive.

The wild-type R. palustris predominately expressed GlnA1, and the deletion of *glnA1* gene dramatically slowed down the rate of growth. Hence, GlnA1 should be primarily responsible for the ammonium assimilation. However, the GlnA1 purified from E. coli cells that were grown under nitrogen-replete conditions just presented a rather low activity. As GlnA1 belongs to GSIs, which are usually dodecamers formed from 2 face-to-face hexagonal rings (14, 15), we speculated that the activity of GlnA1 was inhibited by posttranslational modification. It is already known that the ammonium assimilation catalyzed by GSI is an ATP-dependent reaction. A previous study showed that the adenylylation status of the GS was regulated not only by ammonium availability, but also by light intensity in another photosynthetic diazotroph R. capsulatus, suggesting that intracellular energy state plays an important role in regulating the GS activity (16). To save ATP energy under nitrogen-replete conditions, the activity of GSI is inhibited by covalently adding an AMP to its tyrosine residue at the active center. The GS activity is negatively correlated with the degree of adenylylation of the 12 active centers, which are located between every 2 adjacent monomers of GSIs (17–19). In this study, we found that the adenylylation of residue Tyr^398^ of GlnA1, which was identified via mass spectrometry analysis of the protein purified from R. palustris cells grown with abundant ammonium, caused a significant decrease of its specific activity (Fig. 5 and Fig. S2). The deadenylylation by making a GlnA1^Y398A^ variant or incubating cells under nitrogen-limited conditions obviously increased the specific activity of GlnA1 (Fig. 5), suggesting that the activity of GlnA1 is regulated by the reversible adenylylation/deadenylylation reactions. No adenylylation was detected in GlnA2, GlnA3, and GlnA4 (Table S4 to 6). These data suggest the finely tuned modulation of GS activity by posttranslational modification is just conserved in GSIs.

GS catalyzes the formation of glutamine, which is an important intracellular signaling molecule for nitrogenase regulation in diazotrophs. However, it is still unclear how these GSs influence the expression of Fe-only nitrogenase in R. palustris. Fe-only nitrogenase has recently attracted a great deal of research interest, as it is able to reduce CO_2_ to a powerful greenhouse gas CH_4_ (10). It is already known that R. palustris nifA* mutant generated by deleting 48 bp of the Q-linker region of the transcriptional activator NifA expresses Mo nitrogenase constitutively in ammonium-containing medium (20). However, the nifA* mutant is unable to derepress the expression of Fe-only nitrogenase inhibited by ammonium. Here, we found that R. palustris Δ*glnA1* mutant expressed *anfHDGK* gene cluster and produced H_2_ and CH_4_ when grown with ammonium (Fig. 7). Given that the conversion of CO_2_ to CH_4_ represents a unique feature of Fe-only nitrogenase (10), our results demonstrate that the deletion of *glnA1* could bypass the regulatory networks and activate the expression of Fe-only nitrogenase in the presence of ammonium. Therefore, GlnA1 plays a key role in nitrogenase regulation in response to nitrogen availability.

In addition, GlnA4 showed a high specific activity when it was heterogeneously expressed in E. coli (Fig. 4). However, no obvious expression of *glnA4* was observed in R. palustris grown in PM, and the deletion of *glnA4* did not affect the growth rate of R. palustris. The GSII GlnA2 is the back-up enzyme that is deployed in situations where GlnA1 is absent. However, attempts to obtain a *glnA1 glnA2* double deletion mutant also failed. These data suggest that GlnA4 is unlikely to be responsible for ammonium assimilation as an alternative GS. Its physiological function is still unclear. Future studies will focus on elucidating the role of GlnA4 in nitrogen metabolism.

**FIG 8:**
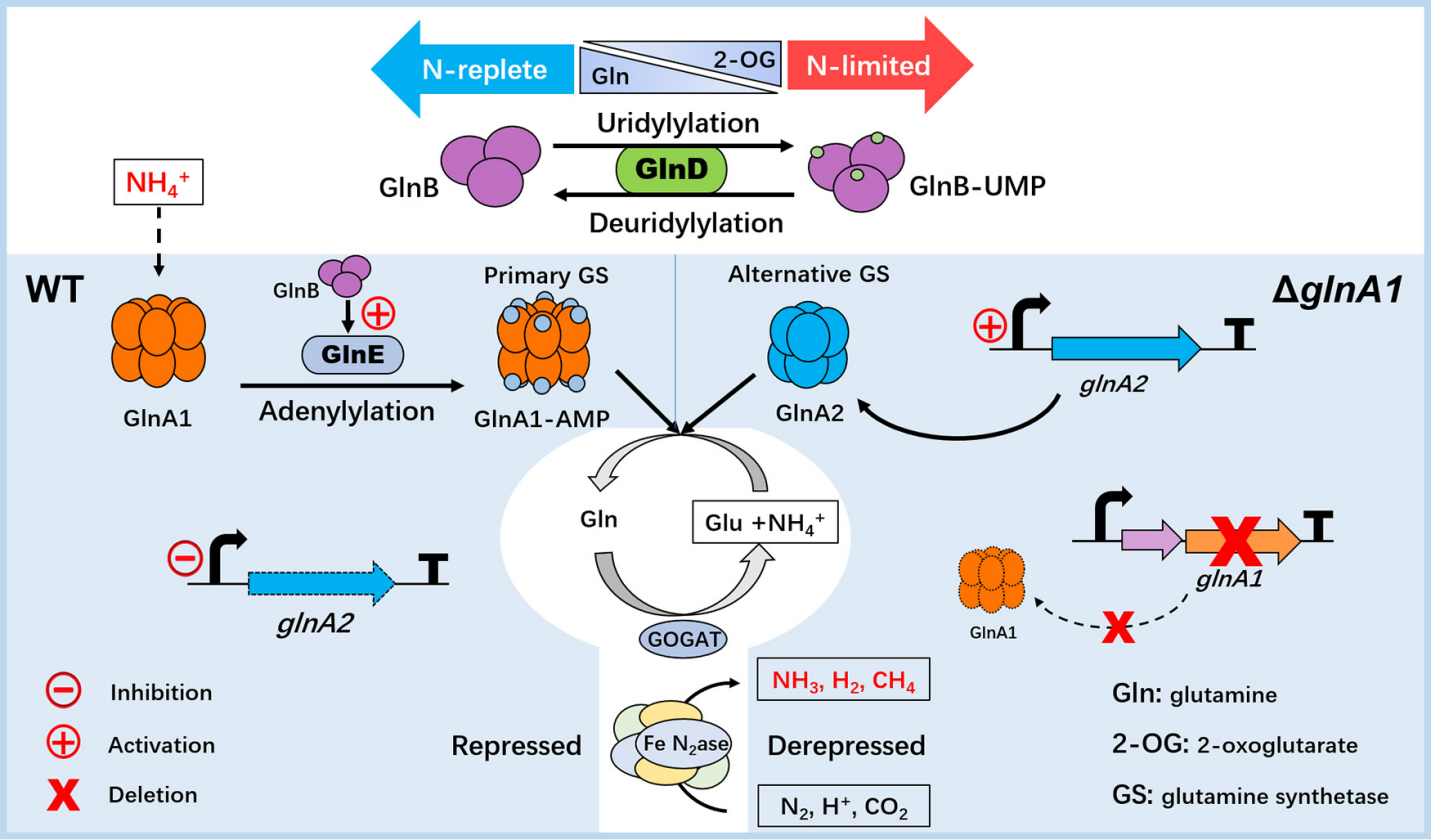
A proposed model for ammonium assimilation and Fe-only nitrogenase regulation in R. palustris. Gln, glutamine; 2-OG, 2-oxoglutarate; GS, glutamine synthetase.

Based on the data we obtained, a model for ammonium assimilation and the regulation of Fe-only nitrogenase in R. palustris was proposed (Fig. 8). In ammonium-grown wild-type cells, the uridylylated GlnB (GlnB-UMP) was deuridylylated by uridylyltransferase (GlnD) (21–23). The adenylyltransferase (GlnE) complex with deuridylylated GlnB stimulates the adenylylation of GlnA1 (GlnA1-AMP), resulting in a significantly decreased activity of GlnA1 to avoid the excess production of glutamine (24, 25). However, the ratio of glutamine to 2-oxoglutarate (Gln/2-OG) is still high enough to repress the expression of the alternative GlnA2 and Fe-only nitrogenase. In contrast, the *glnA1* deficient cells switch to use GlnA2 for ammonium assimilation. Though GlnA2 experiences no regulation of PII proteins but it has a much lower activity than GlnA1. The decreased regeneration rate of glutamine causes a lower ratio of Gln/2-OG and finally results in the expression of Fe-only nitrogenase in the presence of ammonium.

## MATERIALS AND METHODS

### Phylogenetic analysis of GS proteins.

GS proteins were classified based on the phylogenetic relationship with previously reported reference sequences (4). Reference sequences of GS used for evolutionary analysis are summarized in Table S2. Protein sequences were aligned with MUSCLE (26). Evolutionary analyses were conducted in IQ-TREE (27) and visualized using iTOL (28).

### Bacterial strains and growth conditions.

For genetic manipulations, R. palustris strains were grown aerobically on defined mineral medium (PM) agar supplemented with 10 mM succinate at 30°C (29). E. coli was grown in LB medium at 37°C. When appropriate, R. palustris was grown with gentamicin at 100 μg mL^−1^ or kanamycin at 200 μg mL^−1^. E. coli cultures were supplemented with gentamicin at 20 μg mL^−1^ or kanamycin at 50 μg mL^−1^. An anoxic environment of R. palustris cultures was maintained by using sealed culture tubes with N_2_ gas in the headspace. The medium including 20 mM acetate as the carbon source was supplemented with 10 μM sodium molybdate (Na_2_MoO_4_) as indicated. R. palustris cultures grown in the sealed culture tubes were illuminated with a light incubator.

### Genetic manipulation of R. palustris.

All strains, plasmids, and primers used are listed in Table S3. In-frame deletion of the *glnA1* (glnA1-up-F, km-g1-up-R2, g1-km-down-F, glnA1-down-R, km-g1-F, Km-g1-R2), *glnA2* (glnA2-up-F, glnA2-up-R, glnA2-down-F, glnA2-down-R), *glnA3* (glnA3-up-F, glnA3-up-R, glnA3-down-F, glnA3-down-R), or *glnA4* (glnA4-up-F glnA4-up-R, glnA4-down-F, glnA4-down-R) genes was created by PCR using the Q5 high-fidelity DNA polymerase to amplify ~ 1 kb of DNA upstream of the start codon and ~ 1 kb of DNA downstream of the stop codon. These fragments were then incorporated into PstI-digested pJQ200SK suicide vector using the T5 exonuclease-dependent assembly system (30). For the deletion of *glnA1* gene, a kanamycin resistance gene was included in the middle of the 2 homologous fragments. Plasmid pJQ-ΔglnA1::Km^R^, pJQ-ΔglnA2, pJQ-ΔglnA3, and pJQ-ΔglnA4 were mobilized into R. palustris CGA009 by conjugation with E. coli S17-1, and double crossover events for allelic exchange were achieved using a selection and screening strategy as described previously (31). All engineered strains were verified by PCR and sequencing.

### Measurement of cell growth and fluorescence intensity.

Cultures in log phases were harvested by centrifuging at 12,000 rpm for 1 min (Eppendorf Centrifuge 5424R, Eppendorf AG). The cell pellets were washed twice with 10 mM phosphate-buffered saline (PBS). The cells were pelleted by centrifuging at 12,000 rpm for 1 min, and then were resuspended with 10 mM PBS. A 200 μL resuspended culture was then transferred to the 96-well clear and black plates for the measurement of OD_660_ nm and relative fluorescence intensity by a microplate reader (Synergy TMH4, BioTek Instruments), respectively. When measuring the relative fluorescence of RFP, the excitation and emission wavelengths were set to 587 nm and 610 nm, respectively (13).

### Quantitative real-time PCR analysis.

The mRNA expression levels of *anfD* and *glnA* genes were measured by qRT-PCR in mid log phases of R. palustris strains. R. palustris cells were first harvested from cultures grown to the mid log phases. After RNA extraction, cDNA was then prepared with 2 μg total RNA using FastKing-RT SuperMix (TIANGEN Biotech) in a 20 μL reaction mixture, which was incubated at 42°C for 15 min to remove the genomic DNA and complete the reverse transcription. Another incubation at 95°C for 3 min was carried out to stop the enzyme reaction. Reactions were carried out using SuperReal PreMix Plus (TIANGEN Biotech) with 50 ng cDNA and 0.5 μM each primer (Table S3) (q-g1-F/R for *glnA1*, q-g2-F/R for *glnA2*, q-g3-F/R for *glnA3*, q-g4-F/R for *glnA4*, q-anfD-F/R for *anfD*, and q-rpoD-F/R for *rpoD*) in a final volume of 20 μL, with cycling parameters (40 cycles of 95°C for 10 s, 60°C for 60 s) set per optimized cycling conditions for the Applied Biosystems 7500 real-time PCR system (ABI Life Technologies). Transcriptional levels of the genes tested were normalized to the transcriptional levels of the housekeeping gene, *rpoD* (32).

### Protein expression and purification.

The genomic DNA of R. palustris CGA009 was used as template to amplify *glnA1*, *glnA2*, *glnA3*, and *glnA4* genes by PCR using the Q5 high-fidelity DNA polymerase (New England Biolabs) with primers 28a-GlnA1/2/3/4-F/R or 32a-GlnA1/2/3/4-F/R. These PCR fragments were then integrated into the BamHI-digested pET-28a or NcoI-digested pET-32a using the T5 exonuclease-dependent assembly system, respectively. The pET28a-glnA1, pET28a-glnA2, pET28a-glnA3, and pET28a-glnA4 vectors were transformed into E. coli BL21(DE3), and pET32a-glnA1, pET32a-glnA2, pET32a-glnA3, and pET32a-glnA4 were transformed into E. coli BL21-*trxB* (DE3). E. coli cultures carrying the recombinant plasmids were induced by the addition of 0.05 mM isopropyl β-D-1-thiogalactopyranoside (IPTG) and were then incubated at 16°C for 24 h. The harvested cells were resuspended in buffer A (20 mM Tris-HCl, 300 mM NaCl and 20 mM imidazole, pH 7.5), lysed by sonication, and centrifuged at 12,000 rpm for 60 min at 4°C. The collected supernatant was loaded onto an affinity column packed with Ni Sepharose (Cytiva). The resin was washed with buffer A, and the target protein was eluted with 30 to 40% buffer B (20 mM Tris-HCl, 300 mM NaCl, 500 mM imidazole, 1 mM DTT, pH 7.5). The purities of these proteins were checked based on SDS-PAGE analysis with Coomassie staining.

### Determination of specific activities of GS enzymes.

The conversion of l-glutamate to γ-glutamylhydroxamate was used to determine the specific activities of GSs (21). The 200 μL assay mixtures contained 50 mM Tris-HCl (pH 7.5 or as indicated), 10 mM sodium glutamate, 10 mM cysteine, 40 mM hydroxylamine-HCl, 10 mM ATP, 40 mM MgSO_4_, and 1 mM EDTA. After the start of the reaction with purified enzyme, the assay mixtures were incubated for 30 min at 30°C or as indicated. The reactions were then quenched by addition of 100 μL of color developing reagent containing 0.2 M trichloroacetic acid (TCA), 0.37 M ferric trichloride, and 0.6 M hydrochloric acid. After incubation at room temperature for 10 min, the γ-glutamylhydroxamate was quantified by measuring the absorbances at 540 nm.

### Identification of the posttranslational modification of GlnA.

The purified GlnA1, GlnA2, GlnA3, and GlnA4 were digested with trypsin and subjected to an EASY-nLC 1000 interfaced via a Nanospray Flex ion source to an Orbitrap Fusion Tribrid mass spectrometer (Thermo Fisher Scientific) (nano-LC-MS/MS) analysis at the Technological Platform of Mass Spectrum Centre of Institute of Microbiology, Chinese Academy of Sciences. MS/MS data was processed using Mascot search engine (v.2.8.0, 2021, http://www.matrixscience.com; Matrix Science Ltd.). Carbamidomethylation on Cys was specified as fixed modification and oxidation on Met and adenylylation on Tyr were specified as variable modifications. False discovery rate thresholds for protein, peptide and modification site were specified at 1%. All the other parameters in Mascot were set to default values.

### CH_4_ and H_2_ measurements from whole cells.

When cultures of R. palustris strains reached their maximal optical density at 660 nm (OD_660_), gas-phase samples were withdrawn with a Hamilton sample lock syringe from the culture vial headspace. CH_4_ and H_2_ were measured with a Shimadzu GC-2014 gas chromatograph as described previously (33). Total protein concentrations were determined by Bradford assay using bovine serum albumin (BSA) as the standard.
