# Supplementary material for: The Role of Glutamine Synthetase in Regulating Ammonium Assimilation and Iron-Only Nitrogenase Expression in a Photosynthetic Diazotroph
Source: Microbiol Spectr. 2023 Mar 27;11(2):e04953-22. doi: 10.1128/spectrum.04953-22 (PMC10100968; doi:10.1128/spectrum.04953-22)
Supplement: Supplemental file 1 — Supplemental material. Download spectrum.04953-22-s0001.pdf, PDF file, 0.3 MB [file spectrum.04953-22-s0001.pdf]

The role of glutamine synthetase in regulating ammonium assimilation and iron-only nitrogenase expression in a photosynthetic diazotroph

Mingyue Jiang[a,b], Dahe Zhao[a], Lu Huang[a,b], Yan Zeng[a], Jingfang Liu[c], Hua Xiang[a,b], Yanning Zheng[a*]

[a] State Key Laboratory of Microbial Resources, Institute of Microbiology, Chinese Academy of Sciences, Beijing 100101, China;

[b] College of Life Science, University of Chinese Academy of Sciences, Beijing 100049, China

[c] Institutional Center for Shared Technologies and Facilities, Institute of Microbiology, Chinese Academy of Sciences, Beijing 100101, China;

[*] Corresponding author. Mailing address: No.1 Beichen West Road, Chaoyang District, Beijing 100101, China. Tel: +86-10-64806943. E-mail: zhengyn@im.ac.cn.

Table S1 Four glutamine synthetases in *R. palustris* CGA009

Name in this study	GlnA1	GlnA2	GlnA3	GlnA4
Gene number in the genome	RPA2967	RPA4209	RPA1401	RPA0984
Number of amino acids (Daltons)	469	345	461	486
Molecular Weight	52361.67	38676.78	51320.57	54483.1
Number of DNA bases (bp)	1410	1038	1386	1461
classification	I	II	GlnT	GlnT

Table S2 Reference sequences of different types of GS used for evolutionary analysis

Type	Species	Sequence accession
GS I	*Salmonella typhimurium* str. LT2	NP_462887.1
	Escherichia coli str. K-12 substr. MG1655	NP_418306.1
GSII	*Schizosaccharomyces pombe* ASM294v2	NP_593400.1
GSIII	*Chlorobium tepidum* TLS	WP_010933078.1
	Bacteroides thetaiotaomicron ASM1413175v1	WP_008765057.1
	Bacteroides fragilis ASM1688992v1	WP_008768008.1
GlnT	*Sinorhizobium meliloti* 2011	WP_003532529.1

Table S3 Strains, plasmids and primers used in this work

Strain, Plasmid or Primer	Genotype, phenotype or primer sequence	Reference or Source
***E. coli* strains**		
E. coli S17-1	*thi pro hdsR hdsM* + *recA*; chromosomal insertion of RP4-2 (Tc::Mu Km::Tn7)	(1)
E. coli BL21 (DE3)	F^-, ompT, $hsdS_B(r_B^-m_B^-)$, gal, dcm, (DE3)	Invitrogen
E. coli BL21 *trxB* (DE3)	F^-, ompT, $hsdS_B(r_B^-m_B^-)$, gal, dcm, trxB15::kan, (DE3)	Novagen
R. palustris strains		
CGA009	Wild type; *hupV* mutant; spontaneous frameshift (4-bp deletion) in *hupV*	(2)
ΔglnA1::Km^R	CGA009 in which the Km^R was introduced to replace *glnA1* using allelic exchang	This study
ΔglnA2	CGA009 in which the *glnA*2 gene was deleted by allelic exchange	This study
ΔglnA3	CGA009 in which the *glnA3* gene was deleted by allelic exchange	This study
ΔglnA4	CGA009 in which the *glnA4* gene was deleted by allelic exchange	This study
**Plasmids**		
pET28a-glnA1	The *glnA1* gene from *R. palustris* was constructed into *Bam*HI site of pET-28a vector.	This study
pET28a-glnA2	The *glnA2* gene from *R. palustris* was constructed into *Bam*HI site of pET-28a vector.	This study
pET28a-glnA3	The *glnA3* gene from *R. palustris* was constructed into *Bam*HI site of pET-28a vector.	This study
pET28a-glnA4	The *glnA4* gene from *R. palustris* was constructed into *Bam*HI site of pET-28a vector.	This study
pET32a-glnA1	The *glnA1* gene from *R. palustris* was constructed into *Nco*I site of pET-32a vector.	This study
pET32a-glnA2	The *glnA2* gene from *R. palustris* was constructed into *Nco*I site of pET-32a vector.	This study
pET32a-glnA3	The *glnA3* gene from *R. palustris* was constructed into *Nco*I site of pET-32a vector.	This study
pET32a-glnA4	The *glnA4* gene from *R. palustris* was constructed into *Nco*I site of pET-32a vector.	This study

pJQ200SK	Gm^R, *sacB*; mobilizable suicide vector	(3)
pJQ-glnA1::KmR	GmR, in-frame Δ*glnA1*::Km^R cloned into *Pst*I site of pJQ200SK	This study
pJQ-glnA2	GmR, in-frame Δ*glnA2* cloned into *Pst*I site of pJQ200SK	This study
pJQ-glnA3	GmR, in-frame Δ*glnA3* cloned into *Pst*I site of pJQ200SK	This study
pJQ-glnA4	GmR, in-frame Δ*glnA4* cloned into *Pst*I site of pJQ200SK	This study
**Primers**		
32a-GlnA1-F	ACGACGACGACAAGGCCATGACGACCGCTAAAGAAGT	
32a-GlnA1-R	GGATCCGATATCAGCCATGGTTAGTACGAGTAGTACATCTCGAAC	
32a-GlnA2-F	ACGACGACGACAAGGCCATGACCAAGTACAAGCTCGAG	
32a-GlnA2-R	GGATCCGATATCAGCCATGGTCAGGCGGCGGCTGCCG	
32a-GlnA3-F	ACGACGACGACAAGGCCATGCTGAAGACGATCGATACG	
32a-GlnA3-R	GGATCCGATATCAGCCATGGTCAGTAAAGCTCGAAATATTCATTGG	
32a-GlnA4-F	ACGACGACGACAAGGCCATGAACGTGCACCAGCG	
32a-GlnA4-R	GGATCCGATATCAGCCATGGCTAGAACATCTCGAAATATTCGCG	
28a-GlnA1-F	AGCAAATGGGTCGCGGATCCATGACGACCGCT AAAGAAGT	
28a-GlnA1-R	TGTCGACGGAGCTCGAATTCTTAGTACGAGTA GTACATCTCGAAC	
28a-GlnA2-F	AGCAAATGGGTCGCGGATCCATGACCAAGTAC AAGCTCGAG	
28a-GlnA2-R	TGTCGACGGAGCTCGAATTCTCAGGCGGCGGC TGCCG	
28a-GlnA3-F	AGCAAATGGGTCGCGGATCCATGCTGAAGACG ATCGATACG	
28a-GlnA3-R	TGTCGACGGAGCTCGAATTCTCAGTAAAGCTC GAAATATTCATTGG	
28a-GlnA4-F	AGCAAATGGGTCGCGGATCCATGAACGTGCAC CAGCG	
28a-GlnA4-R	TGTCGACGGAGCTCGAATTCCTAGAACATCTC GAAATATTCGCG	
Km-g1-F	TGGTGTCGCCGAAGCGATTAACATGAACGGTACCGAGCTC	

Km-g1-R2	GCAAAGGGGTACTCATGTAACGAGCTCGAATTGGGGATCT
glnA4-down-F	GGCCGCAGAATGTAGCGGTCAGCGGCACCAATTTC
glnA4-down-R	TTGATATCGAATTCCTGCACATGGTGACATCGCCCAGTTTGG
glnA4-up-F	GTGGATCCCCCGGGCTGCAGCGGCATCCTGTGGGCCTATCTCG
glnA4-up-R	CGCTGACCGCTACATTCTGCGGCCGTCAGATGCGG
glnA1-down-R	TTGATATCGAATTCCTGCAGGCAGCCTCCTTTTACGGTCTGGC
g1-km-down-F	GAGCTCGGTACCGTTCATGTTAATCGCTTCGGCGACACCATTC
km-g1-up-R2	AGATCCCCAATTCGAGCTCGTTACATGAGTACCCCTTTGCTACG
glnA1-up-F	GTGGATCCCCCGGGCTGCAGGCGGCCGAGTGCAACTAATTCGG
glnA2-up-F	GTGGATCCCCCGGGCTGCAGGCTCGAAATCGTGATGGCACAGTG
glnA2-up-R	GAGGACCGCATGTGAACCTCCACGGCGACTGCTTG
glnA2-down-F	GTGGAGGTTCACATGCGGTCCTCTCGTTGGAGATG
glnA2-down-R	TTGATATCGAATTCCTGCAGGATGCTGACCTGCGGCCATTATG
glnA3-up-F	GTGGATCCCCCGGGCTGCAGCGAGCATATTCGTCTCTCCGCTC
glnA3-up-R	CCCCGGCCTCACATAGCTGTCGTCCTCACTATAATC
glnA3-down-F	CGACAGCTATGTGAGGCCGGGGTGAAAACGGCTTG
glnA3-down-R	TTGATATCGAATTCCTGCAGCGATCCGCAATGGCAAGCAACGC
q-g1-F	CCTGTCGGAAACCTGCCTC
q-g1-R	GGGACCAGACGCTTGTAGG
q-g2-F	CAACTTCTCCACCGCCTAC
q-g2-R	GTGAGCCGCATGTGGTTGT
q-g3-F4	AATGAACTACACCGATTGCGT
q-g3-R4	TACCACATCGTGTCGCAGAT
q-g4-F3	GCGGATACGCCTTATGAGAC
q-g4-R3	TGCTCCCACTCGGTGACTTC
q-rpoD-F	CGTCCACTCGGTGCAGAAG
q-rpoD-R	GATGTTGCCTTCCTGAATGAG
q-anfD-F	CCAAGGGGACATGGAGAAAGG
q-anfD-F	GGCGTGGGCGTTGAGGTAT

SI References

1. Simon R, Priefer UB, hler A. 1983. A Broad Host Range Mobilization System for In Vivo Genetic Engineering: Transposon Mutagenesis in Gram Negative Bacteria. Nature Biotechnology 1:784-791.
2. Larimer FW, Chain P, Hauser L, Lamerdin J, Malfatti S, Do L, Land ML, Pelletier DA, Beatty JT, Lang AS, Tabita FR, Gibson JL, Hanson TE, Bobst C, Torres JL, Peres C, Harrison FH, Gibson J, Harwood CS. 2004. Complete genome sequence of the metabolically versatile photosynthetic bacterium Rhodopseudomonas palustris. Nat Biotechnol 22:55-61.
3. Quandt J, Hynes MF. 1993. Versatile suicide vectors which allow direct selection for gene replacement in gram-negative bacteria. Gene 127:15-21.

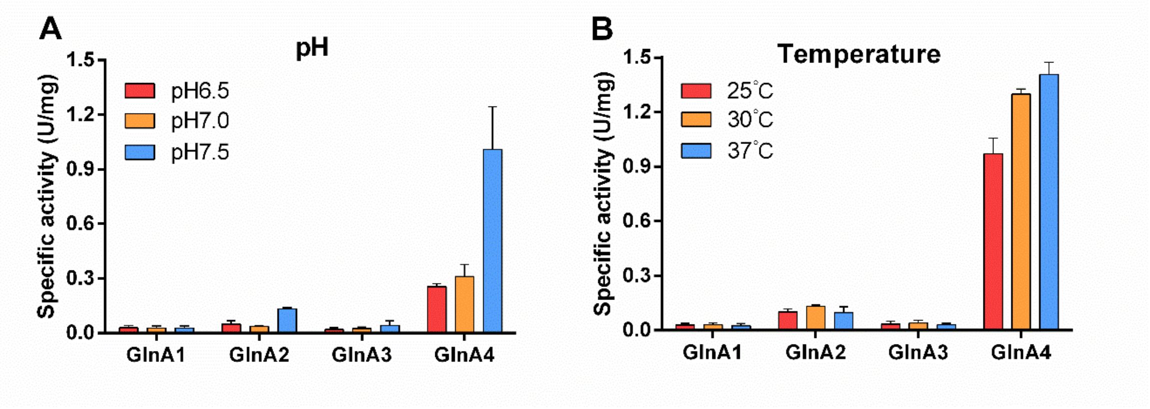

Figure S1 Optimization of enzyme assay conditions for the purified GlnA1, GlnA2, GlnA3 and GlnA4 (GlnA1-A4). (A) The His-tagged GlnA1-A4 fused with thioredoxin (TrxA) exhibited their maximal specific activities at pH 7.5. (B) The His-tagged GlnA1-A4 fused with TrxA showed no obvious difference in specific activity when incubated at 30 °C and 37 °C, respectively.

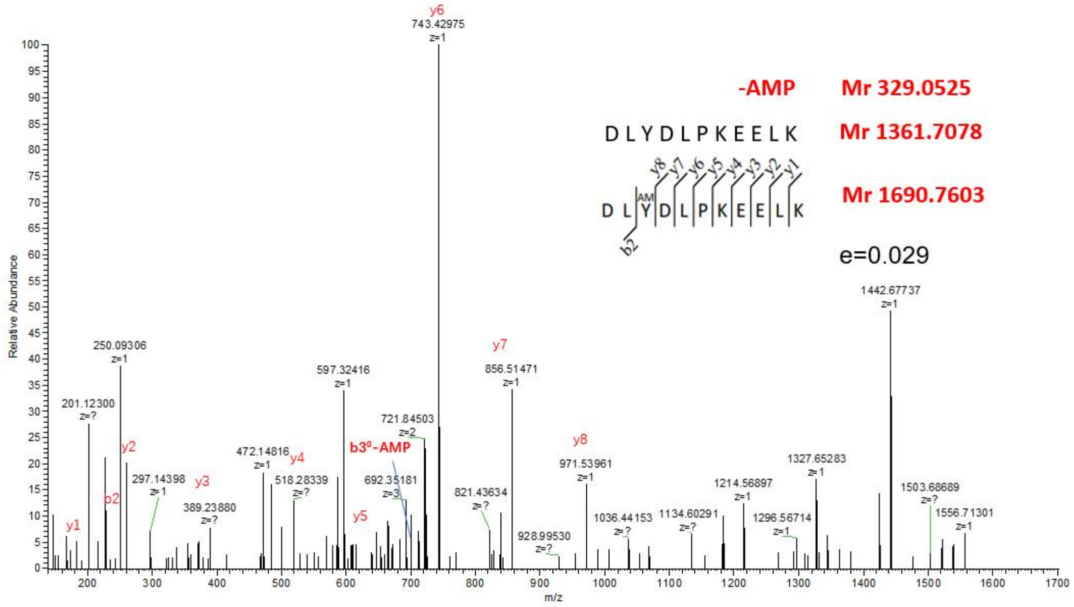

Figure S2 The nano-LC-MS/MS analysis of adenylylation of the purified GlnA1.
